# Non-Enzymatic Amperometric Glucose Sensor Based on Carbon Nanodots and Copper Oxide Nanocomposites Electrode

**DOI:** 10.3390/s20030808

**Published:** 2020-02-02

**Authors:** Tharinee Sridara, Jantima Upan, Gopalan Saianand, Adisorn Tuantranont, Chanpen Karuwan, Jaroon Jakmunee

**Affiliations:** 1 Department of Chemistry, Faculty of Science, Chiang Mai University, Chiang Mai 50200, Thailand; ts.sridara@gmail.com (T.S.); jinny_chem1@hotmail.com (J.U.); 2 The Graduate School, Chiang Mai University, Chiang Mai 50200, Thailand; 3 Faculty of Science, The University of Newcastle, Callaghan, NSW 2308, Australia; saianand.gopalan@gmail.com; 4 National Security and Dual-Use Technology Center, National Science and Technology Development Agency, Pathumthani 12120, Thailand; adisorn.tuantranont@nectec.or.th; 5 Center of Advanced Materials of Printed Electronics and Sensors, Materials Science Research Center, Faculty of Science, Chiang Mai University, Chiang Mai 50200, Thailand; chanpen.karuwan@nectec.or.th; 6 Center of Excellence for Innovation in Chemistry and Research Center on Chemistry for Development of Health Promoting Products from Northern Resources, Faculty of Science, Chiang Mai University, Chiang Mai 50200, Thailand

**Keywords:** non-enzymatic, carbon nanodots, copper oxide, glucose sensor, amperometry

## Abstract

In this research work, a non-enzymatic amperometric sensor for the determination of glucose was designed based on carbon nanodots (C-dots) and copper oxide (CuO) nanocomposites (CuO-C-dots). The CuO-C-dots nanocomposites were modified on the surface of a screen-printed carbon electrode (SPCE) to increase the sensitivity and selectivity of the glucose sensor. The as-synthesized materials were further analyzed for physico-chemical properties through characterization tools such as transmission electron microscopy (TEM) and Fourier-transform infrared spectroscopy (FTIR); and their electrochemical performance was also studied. The SPCE modified with CuO-C-dots possess desirable electrocatalytic properties for glucose oxidation in alkaline solutions. Moreover, the proposed sensing platform exhibited a linear range of 0.5 to 2 and 2 to 5 mM for glucose detection with high sensitivity (110 and 63.3 µA mM^−1^cm^−2^), and good selectivity and stability; and could potentially serve as an effective alternative method of glucose detection.

## 1. Introduction

Diabetes is a disease that occurs when blood glucose level in the body is extremely high, reducing the cells’ ability to absorb sugar and convert it into energy. Normal blood glucose levels in humans should be less than 5.5 mM, with diabetics recording 7.0 mM or more (National Institute for Health and Care Excellence, NICE) [1]. Diabetics are required to check their glucose levels several times a day and take insulin to maintain stability. Hence, the determination of glucose concentration in body fluids is important for the effective diagnosis, monitoring, and treatment of diabetic patients. The challenge in controlling diabetes is strongly associated with the accurate, rapid, and sensitive monitoring of glucose levels and extensive efforts have been devoted to this end in recent years [2,3,4,5].

Glucose biosensors have greatly contributed to the monitoring of glucose levels in diabetic patients [6,7,8,9]. Enzymatic electrochemical biosensors, for example, offer good selectivity and sensitivity [6,10,11,12,13]. However, they also have drawbacks, such as tedious immobilization steps, constrained operational conditions, and poor stability/reproducibility [6,14]. To overcome these shortcomings, enzyme-free (non-enzymatic) glucose biosensing platforms are being designed and developed on the basis of direct oxidation of glucose [15,16,17,18].

Another available and widely used technique for glucose detection is the amperometry. Amperometry offers several advantages, such as ease of use, short analysis time, low detection limit, and lower cost when compared to other sensors [19,20,21]. The traditional amperometric glucose sensor uses an enzyme—glucose oxidase (GOx)—to catalyze the oxidation of glucose and realize high performance (high selectivity/sensitivity). The GOx acts as a catalyst for the oxidation of glucose at the electrode, forming gluconolactone and hydrogen peroxide. The detection of hydrogen peroxide generated at the electrode relates to the concentration of glucose in the sample. However, the enzyme has limitations, such as high cost, poor stability, and low reproducibility. Enzyme activity is also easily affected by temperature, pH, and chemicals [6]. A non-enzymatic sensor, thus, is a suitable alternative, using nanostructured materials that offer enzyme-like activity or nanozymes modified on the surface of the electrode. The nanozymes act as electrocatalysts to generate electrical current by directly oxidizing glucose on the surface of the electrode. They offer several advantages, such as longer storage life, simple modification process, and high selectivity/sensitivity. Therefore, extensive research efforts have been devoted to developing an enzyme-free glucose sensor [22,23,24,25,26]. Non-enzymatic glucose sensors form an oxidation layer, which catalyzes a glucose oxidation reaction, similar to that of the enzyme activity. Various metal nanostructures and their oxides are used as catalysts, including noble metals such as platinum (Pt), gold (Au), and silver (Ag), which exhibit high sensitivity and low detection limits for glucose detection. However, noble metal electrodes have high production costs further limiting their potential applications. Therefore, low-cost materials such as nickel (Ni) and copper (Cu) and their oxides can be used instead [27,28,29,30,31,32]. 

In this work, we used copper oxide (CuO) as a catalyst because of its superior capabilities—low cost, low toxicity, facile preparation, and remarkable electrochemical catalytic property [33,34,35,36]. The electrocatalysis process of glucose oxidation is mediated by Cu(II) in an alkaline solution, which oxidizes into Cu(III) on the surface electrode. Cu(III) is a highly oxidizing state and oxidizes glucose into gluconolactone before reverting back to Cu(II). The current signals acquire electrons by the redox process of Cu(II) and Cu(III) [37,38,39]. Researchers have focused on employing carbon-based nanostructures—such as carbon nanotube (CNT), graphene or graphene oxide (GO)—as supporting materials, with the aim to modify working electrodes, improve sensitivity of the electrode, enhance the surface area, and transfer charge to the nanozyme. Carbon nanodots (C-dots), thus, are a newly emerging class of carbon materials that consist of carbon nanoparticles with diameter less than 10 nm and several hydrophilic surface groups exhibiting excellent water dispersibility, high surface area, and chemical stability [40,41,42]. In addition, a screen-printed carbon electrode (SPCE) has been conveniently used for glucose detection owing to advantages such as flexible design and scope for modification. In this study, a non-enzymatic amperometric glucose sensor based on carbon nanodots (C-dots) and copper oxide (CuO) nanocomposites acting as nanozymes was judicially designed and developed. The as-developed nanocomposites were appropriately characterized and subsequently used to modify SPCE to construct an enzyme-free glucose sensor. The as-constructed non-enzymatic sensing platform possesses unique properties with high active surface area and electrocatalytic activity. In comparison to other materials reported previously, the CuO-C-dots provide primary advantages of improving sensitivity due to the synergistic effects of the nanocomposite (good catalytic activity for glucose oxidation of CuO nanoparticles), increase in surface area of the electrode, and prevention of agglomeration of CuO nanoparticles from C-dots. Besides, the as-developed sensor platform requires facile fabrication and do not involve complicated production processes.

## 2. Materials and Methods

### 2.1. Materials, Chemicals, and Instrumentation

The materials and chemicals used in this work are listed as follows: graphite rod (99.999%, St. Louis, MO, USA, Sigma-Aldrich), ultrapure water (18.2 MΩ cm^−1^), ethanol (99.9%, Bangkok, Thailand, RCl Labscan Limited), sodium hydroxide (Lobachemie, Mumbai, India), copper(II) oxide nanopowder (Sigma-Aldrich, St. Louis, MO, USA), poly-(dimethyldiallylammonium chloride) (PDDA, MW = 200,000–350,000, 20 wt% in water, Sigma-Aldrich, St. Louis, MO, USA), D-glucose (Fisher scientific, New Hampshire, USA), sucrose (Ajax Finechem, Prospect, South Australia), lactose (Fluka, St. Louis, MO, USA), dopamine (Sigma-Aldrich, St. Louis, MO, USA), ascorbic acid (Ajax Finechem, Prospect, South Australia), and uric acid (Sigma-Aldrich, St. Louis, MO, USA). All the chemicals used were of analytical reagent grade.

Transmission electron microscopy (TEM) and scanning electron microscopy (SEM) images were obtained using a transmission electron microscope (JEM 2010, Jeol, Japan) and a field emission scanning electron microscope (JSM 6335 F, Jeol, Japan), respectively. FTIR spectrum was recorded on a FTIR spectrophotometer (Thermo Scientific, Massachusetts, USA) from 400 to 4000 cm^−1^. EmStat potentiostats (PalmSens, Houten, Netherlands) was employed for electrochemical detection.

### 2.2. Synthesis of carbon nanodots (C-dots)

C-dots were synthesized with an electrochemical exfoliation method, using two graphite rods (diameter 3 mm) as the working electrode and the counter electrode placed at a distance of 1.0 cm. Typically, ethanol (140 mL), ultrapure water (10 mL), and NaOH (0.12 g) were mixed to obtain an electrolyte precursor solution. Static potential of 60 V from a direct current (DC) power supply was applied to the two electrodes for 3 h under continuous stirring and N_2_ atmosphere. After 3 h, the excess precipitates were removed by centrifugation at 6000 rpm for 10 min. Consequently, a homogeneous supernatant containing C-dots dispersion was obtained [43,44].

### 2.3. Synthesis of carbon nanodots and copper oxide nanocomposite (CuO-C-dots)

Firstly, 45 mL C-dots and 0.020 g CuO were mixed for 30 min with continuous stirring and the solution was then heated to 100 ˚C. Secondly, 5 mL of 0.5 M NaOH was added to the boiling solution and kept at that temperature for 5 min. After cooling down to room temperature, the suspension was copiously washed with water and ethanol. Finally, the product was subjected to evaporate and CuO-C-dots were collected [45].

### 2.4. Preparation of the CuO-C-dots Nanocomposite Modified Electrode

The prepared 10 mg of CuO-C-dots nanocomposite was dispersed in 1.00 mL of ultrapure water by ultrasonic treatment for 30 min. Then 3 µL of the suspension was dropped on the surface of the SPCE (diameter 2 mm) and dried under an infrared lamp. Finally, 5 µL of 0.5% PDDA solution was coated onto the electrode surface and dried under an incandescent lamp.

### 2.5. Electrochemical Measurement of Glucose

EmStat potentiostats was used for chronoamperometric glucose detection with a three-electrode system using the modified SPCE as a working electrode, carbon as an auxiliary electrode, and Ag/AgCl as a reference electrode using 50 µL of 0.1 M NaOH solution as a total volume of electrolyte solution. The current response was recorded for 100 s for all amperometric experiments. 

## 3. Results and Discussion

### 3.1. Characterizations of C-dots and CuO-C-dots

**Figure 1:**
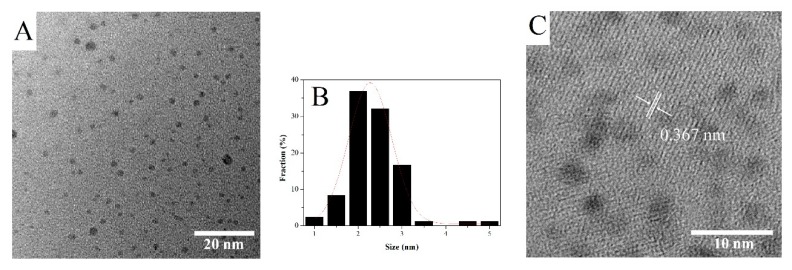
Transmission electron microscopy image (**A**), size distribution histogram (**B**) and high-resolution transmission electron microscopy image (**C**) of the as-synthesized carbon nanodots.

C-dots were synthesized by the electrochemical exfoliation method. A graphite rod acted as the carbon source and alkaline alcohol as an electrolyte. After 3 h, the colorless solution changed into a dark yellow solution, indicating successful preparation of C-dots. The morphology of the synthesized C-dots was further characterized by TEM. It was found that well-dispersed C-dots were of uniform (small spherical) shape with an average diameter of 2 nm, as shown in Figure 1A,B, respectively. Moreover, the lattice fringe of C-dots was analyzed through high-resolution TEM (HRTEM), as shown in Figure 1C. The lattice fringe of about 0.367 nm can be assigned to the (002) reflection plane of graphite [46,47].

**Figure 2:**
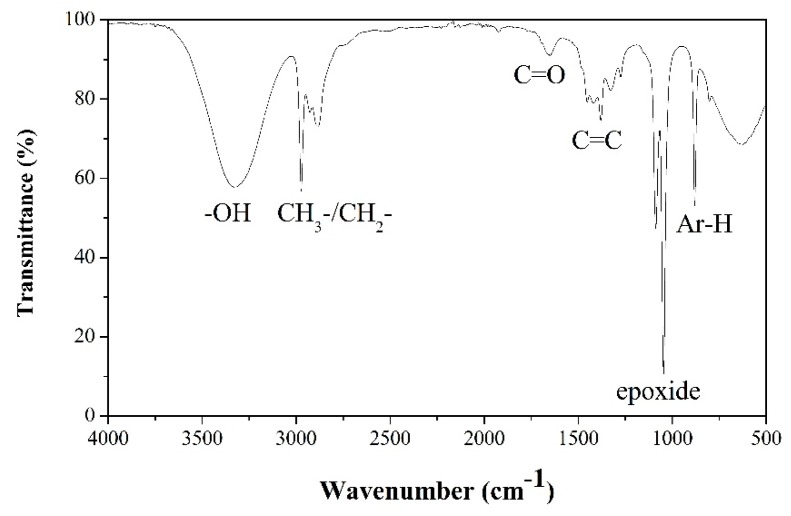
Fourier-transform infrared spectroscopy spectrum of the synthesized carbon nanodots.

FTIR was then used to analyze the functional groups of C-dots, as shown in Figure 2. The presence of the oxygen-containing groups can be confirmed by the stretching vibration bands of broad peak of O−H at 3300 cm^−1^, C=O at 1648 cm^−1^, and the epoxide group at 1087 cm^−1^. Moreover, there were a few absorption peaks, including C−H stretching at 2974 cm^−1^, bending vibrations of aromatic C=C, and aromatic =C−H around 1378 cm^−1^ and 879 cm^−1^, respectively. Owing to the presence of polar functional groups, the synthesized C-dots showed a highly hydrophilic property and excellent dispersibility in water [43,44,48,49].

**Figure 3:**
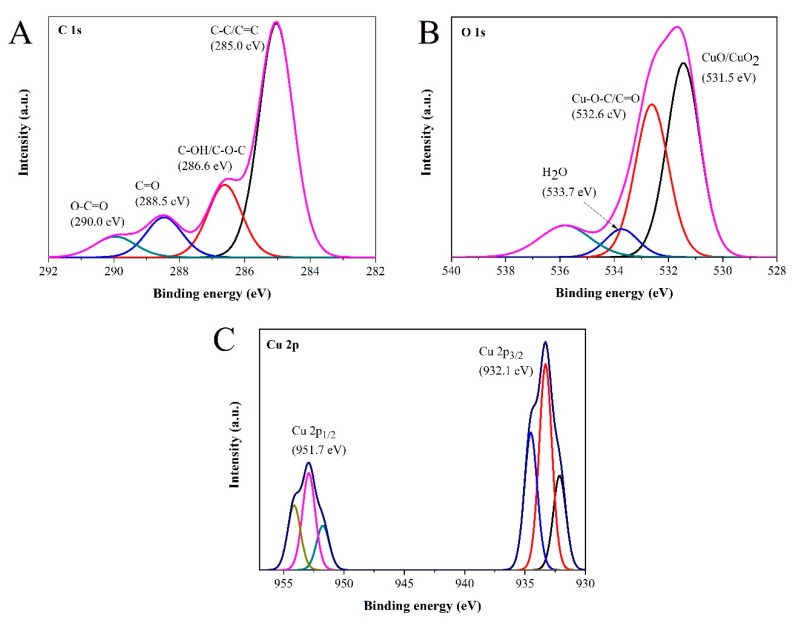
The high-resolution core-level X-ray photoelectron spectroscopy spectra of C 1s (**A**), O 1s (**B**), and Cu 2p (**C**).

X-ray photoelectron spectroscopy (XPS) of the CuO-C-dots nanocomposite was done to further confirm the product’s composition and chemical states. The high-resolution XPS spectrum of C 1s showed the highest intensity, demonstrating the presence of carbon in the nanocomposite structure, as shown in Figure 3A. The main peak at 285.0 eV corresponds to sp^2^ (C-C, C=C) bonding. The shoulders at about 288.5 eV and 290.0 eV correspond to C=O and O-C=O binding energies (BEs), respectively. The BEs for the chemical states of C-OH and C-O-C typically lies at 286.6 eV. The high high-resolution O 1s spectrum in Figure 3B further confirms the presence of CuO and Cu_2_O. The other two peaks are tentatively assigned at 532.6 eV, ascertained by to the presence of Cu-O-C bonding and the C=O groups in C-dots. The BE at 533.7 eV can be attributed to the original oxygen in C-dots and chemisorbed water molecules (H_2_O). Besides, the high-resolution XPS spectrum of Cu 2p shows two characteristic peaks with BEs at 932.1 and 951.7 eV, which can be assigned to Cu 2p_3/2_ and Cu 2p_1/2_, respectively (Figure 3C). The binding energy of the Cu 2p_3/2_ peaks lies in the range of 932.2 eV and 933.3–934.5 eV for Cu^1+^ in Cu_2_O and Cu^2+^ in CuO, respectively, suggesting the presence of CuO in the nanocomposite catalyst. The BEs at 932.1, 933.3, and 954.5 eV correspond to Cu 2p_1/2_ and Cu 2p_3/2,_ respectively, in CuO. The BEs at 951.7, 952.9, and 954.1 eV correspond to Cu 2p_1/2_ and Cu 2p_3/2_ in Cu_2_O, respectively [35,47].

**Figure 4:**
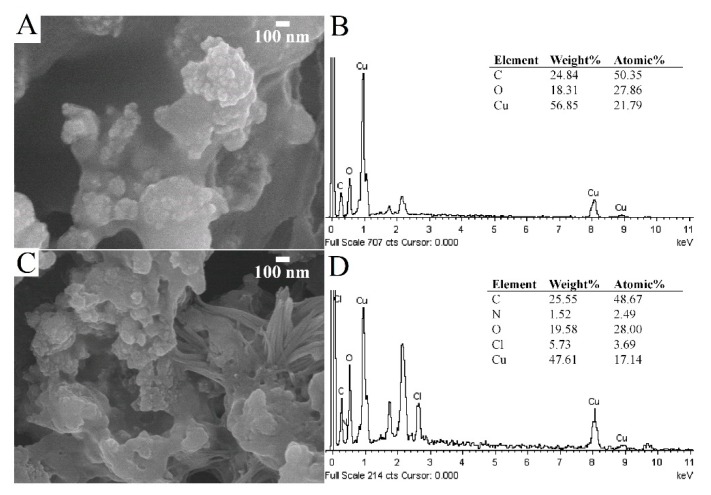
Scanning electron microscopy image (**A**) and its corresponding energy dispersive Spectrometry spectrum (**B**) of the CuO-C-dots on SPCE, and scanning electron microscopy image (**C**), and its corresponding energy dispersive Spectrometry spectrum (**D**) of the PDDA/CuO-C-dots on SPCE.

The nanocomposites of PDDA/CuO-C-dots were then prepared for application as nanozymes for the detection of glucose. The SEM image of the surface of CuO-C-dots modified on SPCE is shown in Figure 4A. It was found that CuO-C-dots were well covered on the electrode surface with larger surface area, and carbon, oxygen, and copper was found in the EDS spectrum, as shown in Figure 4B. In addition, reticular structures were obtained on incorporation of PDDA onto CuO-C-dots, as presented in Figure 4C. The EDS spectrum shown in Figure 4D confirms the existence of carbon, copper, oxygen, nitrogen, and chloride (from PDDA), indicating that the PDDA/CuO-C-dots were successfully modified.

### 3.2. Electrochemical Characterization of the Proposed Electrode

**Figure 5:**
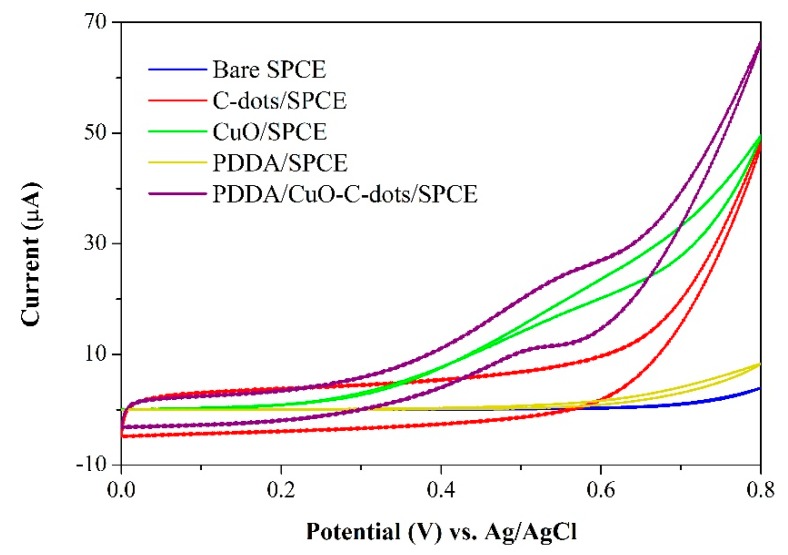
CVs of bare SPCE, C-dots/SPCE, CuO/SPCE, PDDA/SPCE, and PDDA/CuO-C-dots/SPCE for detection of 5 mM glucose in 0.10 M NaOH at a scan rate of 90 mV s^−1^.

Cyclic voltammetry was used to investigate the electrochemical characteristics of glucose oxidation on various modified electrodes in 0.10 M NaOH solution with 5 mM glucose. Cyclic voltammograms are shown in Figure 5. There was no obvious signal response obtained from bare SPCE and PDDA/SPCE. When C-dots were modified on SPCE, a slightly higher background current was observed because of a large surface area of C-dots. Similarly, the current response was significantly increased on the CuO/SPCE, indicating that CuO had ample electrocatalytic activity to glucose oxidation. In addition, the PDDA/CuO-C-dots exhibited a markedly improved current response of glucose oxidation with oxidation peak of about 0.50 V vs. Ag/AgCl. The probable reason for this might be the synergistic effects of nanocomposites, which include (1) good electrical conductivity and high stability of PDDA, (2) good catalytic activity for glucose oxidation of CuO nanoparticles, and (3) enhanced surface area of the electrode and prevention of agglomeration of CuO nanoparticles from C-dots.

The electrocatalytic oxidation of glucose in alkaline solution at the PDDA/CuO-C-dots/SPCE can be described as follows: CuO is firstly oxidized to CuOOH as a strong oxidizing agent. The Cu(III) species then electrochemically oxidized glucose to gluconolactone, responding to the oxidation peak of glucose’s oxidation reaction, as shown in Figure 5 (purple line) [50].

### 3.3. Effect of CuO-C-dots Modified on Screen-printed Carbon Electrode 

**Figure 6:**
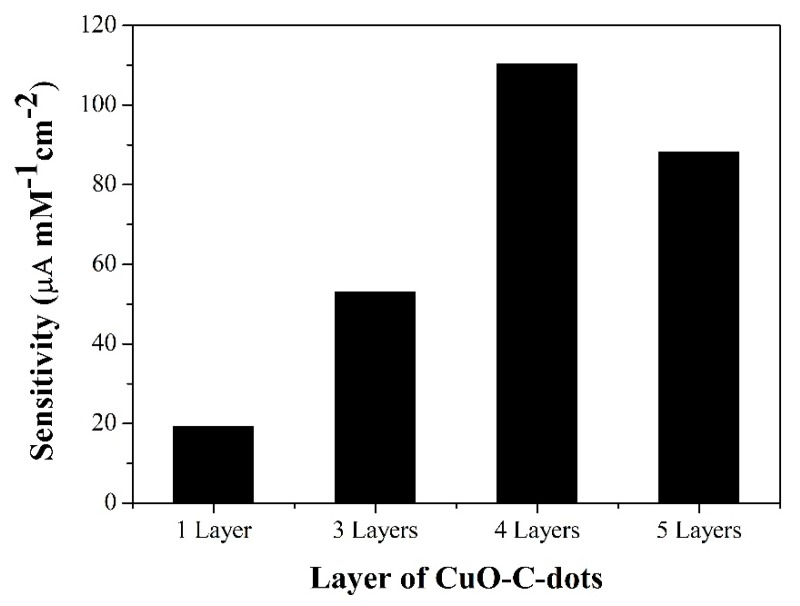
Sensitivity of the electrode achieved with different layers of CuO-C-dots on SPCE for glucose determination in 0.1 M NaOH.

A higher current response of glucose oxidation was obtained from a large catalytic active site on the modified electrode surface. Therefore, the amount of CuO-C-dots nanocomposite was optimized by depositing different layers of CuO-C-dots (each layer is 3 µL of CuO-C-dots suspension) before coating the electrode surface with PDDA. The as-coated multiple layers of suspension were studied by constructing calibration graphs of glucose in a range of 0.5 to 2 mM using amperometric detection at a constant potential of +0.50 V vs. Ag/AgCl; the slopes obtained indicate sensitivity of different layer-modified electrodes. Sensitivity obviously increased with rise in CuO-C-dots layer up to four layers and decreased after continuous deposition, as shown in Figure 6. Thick films can hinder electron transfer between glucose and the electrode surface, and reduce the electrocatalytic active area of the modified electrode. Thus, four sequential layers of CuO-C-dots were selected for further study.

### 3.4. Amperometric Detection of Glucose by the Proposed Electrode

**Figure 7:**
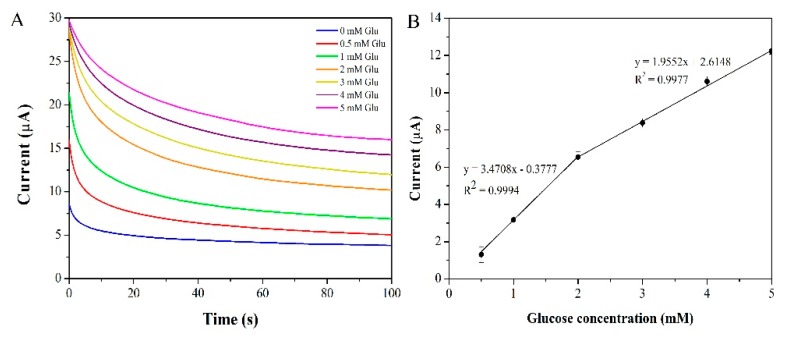
The chronoamperometric responses of PDDA/CuO-C-dots/SPCE for various glucose concentrations (0.5–5 mM) in 0.1 M NaOH at +0.50 V (**A**) and their calibration curve of current response vs. glucose concentration (**B**).

Chronoamperometry was employed as a detection technique to determine glucose using PDDA/CuO-C-dots/SPCE as a working electrode at a constant oxidation potential of +0.50 V. The steady current from 80–100 s was used as a current response, and current increased with increasing glucose concentration, as shown in Figure 7A. The calibration graph in Figure 7B shows two linear ranges: 0.5 to 2 mM and 2 to 5 mM with a limit of detection of 0.2 mM. The current began to saturate at a glucose concentration higher than 5 mM due to limitations of the active surface of the modified electrode. Their corresponding linear equations were I (µA) = 3.4708C (mM) + 0.3777 with R^2^ = 0.9994 and I (µA) = 1.9552C (mM) + 2.6148 with R^2^ = 0.9977, respectively. It was found that sensitivity at high concentration was lower than that at low concentration due to the saturated adsorption dynamics of the former. A summary of the analytical performance of various glucose sensors, including enzymatic and non-enzymatic ones, is shown in Table 1. The developed non-enzymatic glucose sensor exhibited good analytical characteristics such as good linearity and high sensitivity. Moreover, the preparation and detection procedures of the proposed method were also simple, quick, and cost-effective.

### 3.5. Selectivity and Stability of the Glucose Sensor

**Figure 8:**
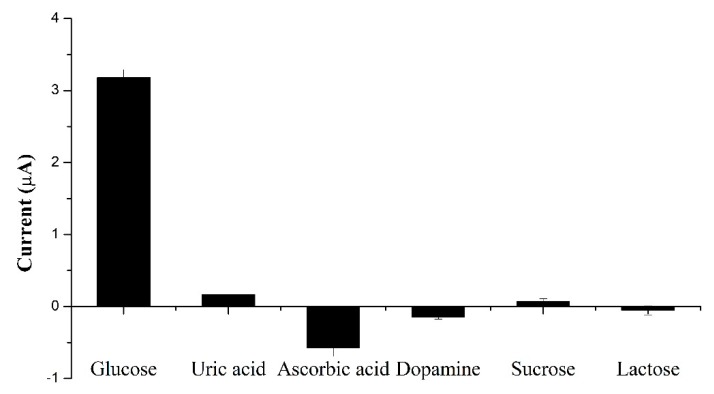
Amperometric response of the proposed sensor to interfering species.

The physiological level of glucose concentration is normally about 3–8 mM, which is higher than that of main interferences such as ascorbic acid (0.1 mM) and uric acid (0.1 mM) [56]. Therefore, the amperometric responses of the proposed sensor with 1 mM glucose and other interfering species (ascorbic acid, uric acid, dopamine, sucrose, and lactose) at a concentration of 0.1 mM were investigated. Figure 8 shows a negligible current response of other species, compared to glucose, demonstrating that the fabricated sensor had excellent selectivity toward glucose.

Furthermore, the stability of the PDDA/CuO-C-dots/SPCE was studied by measuring the current response of 5 mM glucose by storing the modified electrode at room temperature. It was found that there was no obvious change in current response after 12 days, thus highlighting good stability of the proposed sensor. Batch variation of the CuO-C-dots-based amperometric sensor was evaluated. The repeatability of the modified electrode was found to be 2.6% RSD (n = 7).

### 3.6. Applicability of the Prepared Sensor for Glucose Measurement in Real Time 

To evaluate the practical usefulness of the as-fabricated sensor, the proposed sensor was used to determine glucose levels in human blood serum samples. Although many proteins in serum samples can act as insulators and block electron transfer between the solution and the electrode surface, the serum samples should be significantly diluted (100 times with 0.1 M NaOH solution) before analysis. The samples were spiked with standard glucose to obtain final concentrations at 0.5, 1.0, and 3.0 mM. The recoveries of the spiked samples were found from 88 to 94%, as shown in Table 2. The analytical results indicate that the PDDA/CuO-C-dots nanocomposite modified on SPCE is reliable for determining glucose, and nanocomposites could be a possible alternative nanozyme in constructing new kind of glucose sensors. However, to enhance the applicability of the sensor for low-level detection, sample preparation should be optimized to further separate other type of interferences.

## 4. Conclusions

In summary, we successfully prepared a CuO-C-dots nanocomposite and fabricated a non-enzymatic glucose sensor based on PDDA/CuO-C-dots, modified on an SPCE electrode. Due to the synergistic effects of the high surface area of C-dots, good electrical conductivity of PDDA, and remarkable catalytic active sites of CuO nanoparticles, the as-prepared nanocomposites showed excellent electrochemical properties in catalyzing the oxidation reaction of glucose. The developed sensor (CuO-C-dots/SPCE) offers several advantages, which include simple procedures for preparation and detection, fast analysis, high sensitivity, and good selectivity and stability. Moreover, the satisfied recoveries indicate that the sensor is reliable and can be applied to the determination of glucose in real samples.

## Figures and Tables

**Table 1:** Comparison of the analytical performance of different glucose-sensing electrodes.

Sensing Electrode	Detection Method	Potential (V)	Sensitivity (µA mM^−1^cm^−2^)	Linear Range (mM)	LOD (mM)	Ref.
GOx/CdS/Gr on GCE	CV	-	1.76	2–16	0.7	[51]
PDDA/Ch/GOx/PtAuNPs/PtZn on Pt	Amp	+0.60	17.85	0.01–8	0.001	[52]
Au/GO on GCE	Amp	+0.0	5.20, 4.56	0.1–2, 2–16	0.025	[53]
Cu/Cu_2_O/CSs on GCE	Amp	+0.65	63.8, 22.6	0.01–0.69, 1.19–3.69	0.005	[54]
Nafion/NPC-CB on GCE	Amp	+0.64	33.75	0.006–3.369	0.002	[55]
PDDA/CuO-C-dot on SPCE	Amp	+0.50	110, 62.3	0.5–2, 2–5	0.2	This work

Amp = amperometry, Gr = graphene, GCE = glassy carbon electrode, PtAuNPs = platinum and gold nanoparticles, GO = graphene oxide, CSs = carbon spheres, and NPC-CB = nanoporous copper and carbon back

**Table 2:** Determination of glucose in human blood serum samples.

Sample	Spiked Glucose (mM)	Detected Glucose (mM)	%Recovery
1	0.5	0.47 ± 0.05	94
2	1.0	0.88 ± 0.08	88
3	3.0	2.71 ± 0.26	90
